# Mathematical analysis of a measles transmission dynamics model in Bangladesh with double dose vaccination

**DOI:** 10.1038/s41598-021-95913-8

**Published:** 2021-08-16

**Authors:** Md Abdul Kuddus, M. Mohiuddin, Azizur Rahman

**Affiliations:** 1 Australian Institute of Tropical Health and Medicine, James Cook University, Townsville, QLD Australia; 2 Department of Mathematics, University of Rajshahi, Rajshahi, 6205 Bangladesh; 3 Department of Mathematics, Comilla University, Cumilla, 3506 Bangladesh; 4 School of Computing and Mathematics, Charles Sturt University, Wagga Wagga, NSW 2678 Australia

**Keywords:** Computational biology and bioinformatics, Diseases, Mathematics and computing

## Abstract

Although the availability of the measles vaccine, it is still epidemic in many countries globally, including Bangladesh. Eradication of measles needs to keep the basic reproduction number less than one $(\mathrm{i}.\mathrm{e}. \, \, {\mathrm{R}}_{0}<1)$. This paper investigates a modified (SVEIR) measles compartmental model with double dose vaccination in Bangladesh to simulate the measles prevalence. We perform a dynamical analysis of the resulting system and find that the model contains two equilibrium points: a disease-free equilibrium and an endemic equilibrium. The disease will be died out if the basic reproduction number is less than one $(\mathrm{i}.\mathrm{e}. \, \, {\mathrm{ R}}_{0}<1)$, and if greater than one $(\mathrm{i}.\mathrm{e}. \, \, {\mathrm{R}}_{0}>1)$ epidemic occurs. While using the Routh-Hurwitz criteria, the equilibria are found to be locally asymptotically stable under the former condition on ${\mathrm{R}}_{0}$. The partial rank correlation coefficients (PRCCs), a global sensitivity analysis method is used to compute ${\mathrm{R}}_{0}$ and measles prevalence $\left({\mathrm{I}}^{*}\right)$ with respect to the estimated and fitted model parameters. We found that the transmission rate $(\upbeta )$ had the most significant influence on measles prevalence. Numerical simulations were carried out to commissions our analytical outcomes. These findings show that how progression rate, transmission rate and double dose vaccination rate affect the dynamics of measles prevalence. The information that we generate from this study may help government and public health professionals in making strategies to deal with the omissions of a measles outbreak and thus control and prevent an epidemic in Bangladesh.

## Introduction

Many people are being infected every year by serious respiratory infectious diseases, including measles. A significant number of them die or suffer severe illness and life-long complications^1–5^. The annual reports on the estimated number of measles cases and measles caused deaths worldwide are announced by the World Health Organization (WHO) and UNICEF based on the reported data of the member countries. According to WHO and UNICEF statistics for 2017, the total measles cases and measles-related deaths were recorded at 7,585,900 and 124,000, respectively. In 2018, there were about 9,769,400 recorded measles cases and 142,300 measles caused deaths^6^. The maximum cases were reported from Madagascar, Ukraine, Somalia and Liberia. Also, some developed countries, including the United Kingdom, Greece, Czechia, and Albania, lost the elimination status of measles in the latest year. Moreover, the United States counted the maximum number of cases, which was highest in 25 years, in 2018. In 2019, the maximum number of 207,500 people died due to measles, and the reported measles cases were 869,770 globally^7^. This year, Madagascar, Ukraine and Congo have reported the highest numbers of cases. Outbreaks are continuous in Angola, Cameroon, Kazakhstan, Chad, Nigeria, Thailand, Philippines, South Sudan and Sudan^8^. These continuous annually increments are indicating a matter of concerning issue in the world.

Measles is one of the most contagious respiratory infectious diseases caused by the measles virus that lives in an infected person’s nose and throat mucus. It is a virus of paramyxovirus family, genus morbilivirus, and this virus is found only in the human body among all animal species^1,2^. This virus can be spread directly from person to person through coughing and sneezing of the infected person. The clinical symptoms of measles are high fever, runny nose, cough, conjunctivitis, rhinitis, small white spots and a rash in the body of the infected people. This disease is more dangerous, especially for children under five years of age and adults older than 20 years of age. The complications including pneumonia, mouth ulcer, sinus and ear infections, diarrhea, malnutrition, blindness and brain damage may occur due to measles^3^. There is no specific medicine for the treatment of infected people with measles. According to the complications of the patients, a specific treatment may be suggested. Patients may need complete bed rest, fluids, control of fever and pains, and antibiotics^9^. Now, the measles vaccine is available, which is effective and inexpensive, and it has been possible to remarkably reduce the number of people dying from measles through vaccination^5^. The measles, mumps, and rubella (MMR) vaccines efficacy is 95% for preventing measles if the first dose is given to children at 12 months of age, and the efficacy increase to 99% after the second dose is given to children at greater than 12 months of age^10^.

Many countries and various public health organizations such as WHO, UNICEF, American Red Cross, Centers for Disease Control and Prevention (CDC), and United Nations Foundation have conducted tremendous efforts worldwide to fight against measles. These organizations launched the Measles and Rubella Initiative (MRI), a global partnership among these organizations to stop measles and rubella, in 2001 to reduce measles deaths globally by 90% by 2010 compared to 2000 estimates^11^. Presently, this partnership has taken the Measles and Rubella Strategic Framework 2021–2030 (MRSF 2021–2030) for a world free from measles and rubella^12^. However, despite being vaccine-preventable, measles is still a public health problem in many developing countries globally, especially in parts of Asia and Africa, because of low awareness, civil strife, vaccine hesitancy, lower immunization system and poor health infrastructures^13^.

In Bangladesh, one of the South East Asia Region (SEAR) countries, measles outbreaks occurred several times in different areas during 2000–2016. There were about 70,273 reported measles cases and 33,213 confirmed measles cases in Bangladesh during this period^14^. Although the Expanded Program of Immunization (EPI) was started in Bangladesh in 1979 to control and prevent measles^15^, the government adopted more initiatives like strengthening the surveillance system and introduction of the second dose of measles-containing vaccine (MCV2) in 2014 to eliminate measles from the country by 2018^16^. Consequently, measles cases were reduced up to 84% over the last decades. However, the number of estimated measles cases has been increasing since 2016 nationwide. Despite some existing challenges, Rohingya refugees is another challenge for the removal of measles from Bangladesh.

In recent decades, the research relating to measles in epidemiology has been one of the most important research fields to researchers. Many researchers have already proposed their ideas and accomplished their research mathematically, theoretically or experimentally, using different deterministic or compartmental models, to find the comparatively best ways for measles control and prevention, focusing on different areas of the world, for example, London^17^, Afghanistan^18^, Kenya^19^, Madagascar^20^, Ontario^21^, Cape Coast^22^, Italy^23^, Senegal^23^, Taiwan^24^ and China^25,26^. Moreover, Momoh et al.^27^ studied an SEIR deterministic epidemic model to investigate the impact of asymptomatic individuals at the latent period on measles dynamics. Adewale et al.^28^ developed a mathematical model to ascertain the effect of distance between infected and non-infected persons in controlling the measles virus transmission. They observed that the number of infected individuals decreases due to increases in distance between infected and susceptible persons. Also, two studies highlighted the efficiency of vaccination in controlling and prevention of measles transmission^29,30^. Garba et al.^31^ also studied a compartmental mathematical model to examine the effect of vaccination and treatment on measles dynamics. Beay^4^ proposed a SIQR epidemic model and accomplished the numerical analysis of the model to explore the effect of treatment and quarantine on measles dynamics. The study demonstrated that the combined application of quarantine and treatment is more effective to control and prevent measles. It also observed that the measles spread reduces due to the treatment and quarantine of infected individuals.

In this study, we develop a novel compartmental measles model to simulate the prevalence of measles estimation in Bangladesh. We use the next-generation matrix method to determine the basic reproduction number of the system and found that this is an essential determinant for disease dynamics. To supplement and validate the analytic process, we use numerical techniques to solve the model equations and explore the epidemic trajectory for a range of possible parameters values and initial conditions. The local stability analyses of the disease-free and endemic equilibria are examined using the Routh-Hurwitz criteria. Following this, we perform a sensitivity analysis to investigate the model parameters that greatly influence measles prevalence. Finally, we investigate the impact of progression rate, transmission rate and double dose vaccinations on the dynamics of the measles outbreak.

## Methods and materials

### Model description

We developed a compartmental transmission dynamics measles model between the following mutually exclusive compartments: susceptible individuals, $\mathrm{S}(\mathrm{t})$; those who have not yet infected with the disease but might become infected; first dose vaccinated individuals, ${\mathrm{V}}_{1}(\mathrm{t})$; those who have received the first dose of vaccine; second dose vaccinated individuals, ${\mathrm{V}}_{2}(\mathrm{t})$; those who have received the second dose of vaccine; Exposed individuals, $\mathrm{E}(\mathrm{t})$; representing those that are infected and have not yet developed active measles disease; Infected individuals, $\mathrm{I}(\mathrm{t})$; those who are infected and infectious; and Recovered individuals, $\mathrm{R}(\mathrm{t})$; those who were previously infected and successfully recovered. Individuals in the recovery class are neither infectious nor susceptible, including people in treatment, isolation, no longer contacting others or dead.

The total population size $\mathrm{N}(\mathrm{t})$ is assumed to be constant and well mixed:

1 $$\mathrm{N}(\mathrm{t})=\mathrm{S}(\mathrm{t})+{\mathrm{V}}_{1}(\mathrm{t})+{\mathrm{V}}_{2}(\mathrm{t})+\mathrm{E}(\mathrm{t})+\mathrm{I}(\mathrm{t})+\mathrm{R}(\mathrm{t}).$$

**Figure 1:**
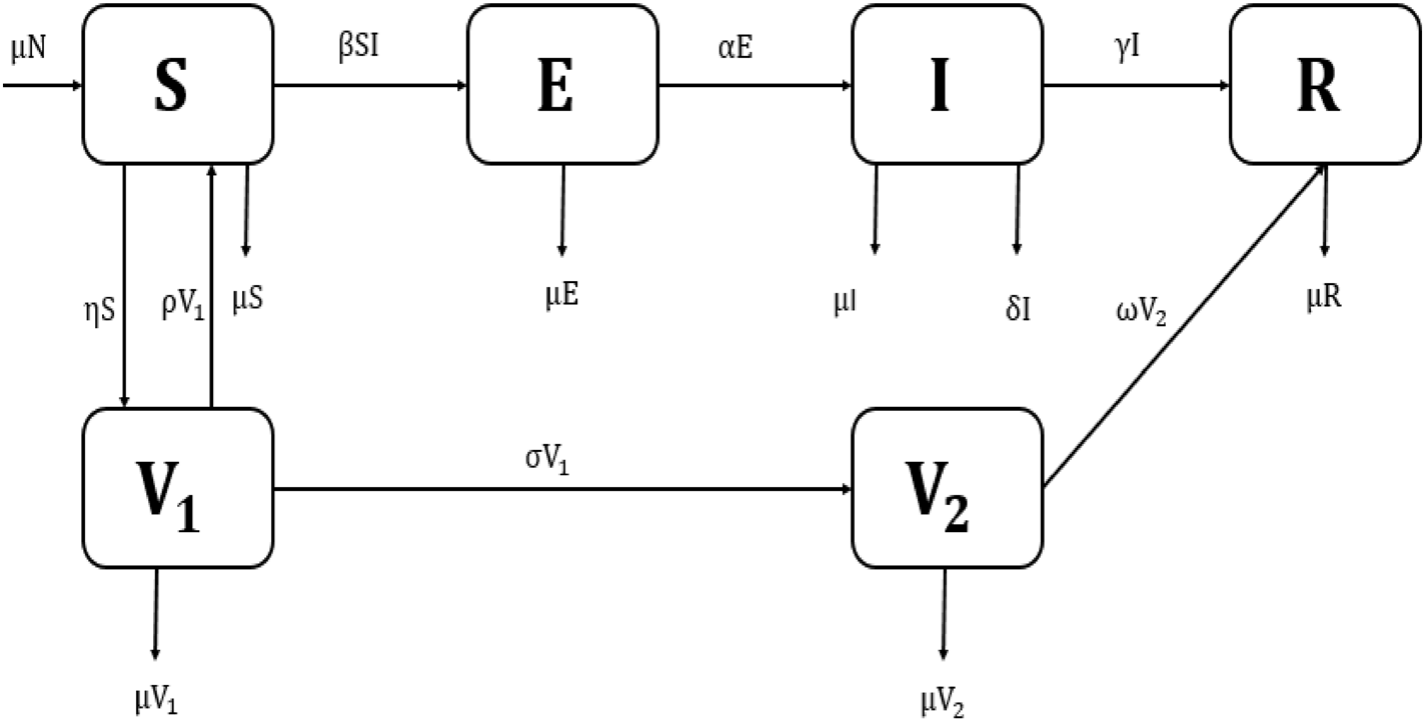
Schematic diagram of measles model for Bangladesh measles setting.

To ensure the population size constant, we replace all deaths as newborns in the susceptible compartment. It includes death through natural causes, which occurs in all states at the constant per-capita rate $\upmu$, and measles-related deaths, which occur at the constant per capita rate $\updelta$. Susceptible population $(\mathrm{S})$ who receive the first dose of vaccine move to the vaccinated compartment at a rate $\upeta$. The first dose of vaccinated population ${\mathrm{V}}_{1}$ moves to the susceptible compartment at a rate $\uprho$, and the rest of the population moves to the second dose of vaccinated population ${\mathrm{V}}_{2}$ at a per-capita rate $\upsigma$. The second dose of the vaccinated population also moves to the recovery compartment at a rate $\upomega$. Individuals in the $\mathrm{S}$ compartment may be infected with the measles virus at a rate $\uplambda =\mathrm{\upbeta SI}$, where $\upbeta$ is the transmission rate between infected and susceptible population. Then infected individuals move to the exposed compartment $\mathrm{E}$. A proportion of the exposed population progress to the infected compartment at a per-capita rate $\mathrm{\upalpha }$. A proportion of the infected individuals move to the recovery compartment due to the treatment and natural recovery rate $\upgamma$. The model flow diagram is presented in Fig. 1.

From those as mentioned above, the transmission dynamics of measles is given by the following compartmental system of nonlinear ordinary differential equations that describe the model:

2 $$\frac{\mathrm{dS}}{\mathrm{dt}}={\upmu \mathrm{N}}-\mathrm{\upbeta SI}-\mathrm{\upeta S}-{\upmu S}+\uprho {\mathrm{V}}_{1},$$

3 $$\frac{{\mathrm{dV}}_{1}}{\mathrm{dt}}=\mathrm{\upeta S}-\uprho {\mathrm{V}}_{1}-\upsigma {\mathrm{V}}_{1}-\upmu {\mathrm{V}}_{1},$$

4 $$\frac{{\mathrm{dV}}_{2}}{\mathrm{dt}}=\upsigma {\mathrm{V}}_{1}-\upomega {\mathrm{V}}_{2}-\upmu {\mathrm{V}}_{2},$$

5 $$\frac{\mathrm{dE}}{\mathrm{dt}}=\mathrm{\upbeta SI}-\mathrm{\upalpha E}-{\upmu E},$$

6 $$\frac{\mathrm{dI}}{\mathrm{dt}}=\mathrm{\upalpha E}-\mathrm{\upgamma I}-\mathrm{\delta I}-{\upmu I},$$

7 $$\frac{\mathrm{dR}}{\mathrm{dt}}=\mathrm{\upgamma I}+\upomega {\mathrm{V}}_{2}-{\upmu R}.$$

The initial conditions of the system (2)–(7) are of the form.

8 $$\mathrm{S}\left(0\right)\ge 0, {\mathrm{V}}_{1}\left(0\right)\ge 0, {\mathrm{V}}_{2}\left(0\right)\ge 0,\mathrm{ E}\left(0\right)\ge 0,\mathrm{ I}\left(0\right)\ge 0,\mathrm{ R}(0)\ge 0.$$

It can be easily shown that the solution of the system (2)–(7) subject to the initial conditions (8) exists and is nonnegative for all $\mathrm{t}\ge 0$.

Given the non-negative initial conditions of the system (2)–(7), it is direct to show that each state variable remains non-negative. Summing Eqs. (2)–(7), we find that the total population, $\mathrm{N}(\mathrm{t})$ satisfies in the absence of death due to measles or if there are no infected individuals (i.e. I = 0)^32^, then we have

$$\frac{\mathrm{dN}}{\mathrm{dt}}=\frac{\mathrm{dS}}{\mathrm{dt}}+\frac{{\mathrm{dV}}_{1}}{\mathrm{dt}}+\frac{{\mathrm{dV}}_{2}}{\mathrm{dt}}+\frac{\mathrm{dE}}{\mathrm{dt}}+\frac{\mathrm{dI}}{\mathrm{dt}}+\frac{\mathrm{dR}}{\mathrm{dt}}=0,$$

Integrating this equation, we find

$$\mathrm{N}\left(\mathrm{t}\right)=\mathrm{Constant}.$$

Given the constant population size and positivity of solutions, it naturally follows that each states $\mathrm{S}, {\mathrm{V}}_{1}, {\mathrm{V}}_{2},\mathrm{ E},\mathrm{ I},\mathrm{ R}$ are bounded.

Note that Eqs. (2)–(6) are independent of the recovery population; hence if we only wish to track measles incidence and prevalence, we can focus our attention on the following system:

9 $$\frac{\mathrm{dS}}{\mathrm{dt}}={\upmu \mathrm{N}}-\mathrm{\upbeta SI}-\mathrm{\upeta S}-{\upmu S}+\uprho {\mathrm{V}}_{1},$$

10 $$\frac{{\mathrm{dV}}_{1}}{\mathrm{dt}}=\mathrm{\upeta S}-\uprho {\mathrm{V}}_{1}-\upsigma {\mathrm{V}}_{1}-\upmu {\mathrm{V}}_{1},$$

11 $$\frac{{\mathrm{dV}}_{2}}{\mathrm{dt}}=\upsigma {\mathrm{V}}_{1}-\upomega {\mathrm{V}}_{2}-\upmu {\mathrm{V}}_{2},$$

12 $$\frac{\mathrm{dE}}{\mathrm{dt}}=\mathrm{\upbeta SI}-\mathrm{\upalpha E}-{\upmu E},$$

13 $$\frac{\mathrm{dI}}{\mathrm{dt}}=\mathrm{\upalpha E}-\mathrm{\upgamma I}-\mathrm{\delta I}-{\upmu I}.$$

Given the positivity and boundedness of the system solutions, we find that the feasible region for Eqs. (9)–(13) given by.

14 $$\mathrm{D}=\left\{\left(\mathrm{S}, {\mathrm{V}}_{1}, {\mathrm{V}}_{2},\mathrm{ E},\mathrm{ I}\right)\in {\mathbb{R}}_{+}^{5}:\mathrm{S}+{\mathrm{V}}_{1}+{\mathrm{V}}_{2}+\mathrm{E}+\mathrm{I}=\mathrm{N}\right\}.$$

where D is the positively invariant region for the system (9)–(13). Therefore, in this study, we consider Eqs. (9)–(13) in the set $\mathrm{D}$.

### Ethical approval

This study is based on aggregated measles surveillance data in Bangladesh provided by the World Health Organization. No confidential information was included because mathematical analyses were performed at the aggregate level.

## Results

### Existence of equilibria

Two types of equilibrium solutions appear in this system: the disease-free equilibrium, which is reached when the basic reproduction number is less than one, i.e. ${\mathrm{R}}_{0}<1$ and the endemic equilibrium, which is reached when the basic reproduction number is greater than one, i.e. ${\mathrm{R}}_{0}>1$. We discuss these in order below.

### Disease-free equilibrium point $({\mathbf{X}}^{0})$

In this section, we obtain the disease-free equilibrium point of the system (9)–(13) at which the epidemic is eliminated by applying $\mathrm{E}=\mathrm{I}=0$. Hence, the disease-free equilibrium point is given by

$${\mathrm{X}}^{0}=\left({\mathrm{S}}^{0}, {\mathrm{V}}_{1}^{0}, {\mathrm{V}}_{2}^{0}, {\mathrm{E}}^{0}, {\mathrm{I}}^{0}\right) =\left(\frac{{\upmu \mathrm{N}}(\uprho +\upsigma +\upmu )}{\left(\upeta +\upmu \right)\left(\uprho +\upsigma +\upmu \right)-\mathrm{\uprho \upeta }}, \frac{{\upmu \upeta \mathrm{N}}}{\left(\upeta +\upmu \right)\left(\uprho +\upsigma +\upmu \right)-\mathrm{\uprho \upeta }}, \frac{{\upmu \upeta \sigma \mathrm{N}}}{(\upomega +\upmu )\left(\left(\upeta +\upmu \right)\left(\uprho +\upsigma +\upmu \right)-\mathrm{\uprho \upeta }\right)}, 0, 0\right).$$

That describes the state in which there is no infection in the community, and the total population $\mathrm{N}$ is constant at time $\mathrm{t}=0$.

### Basic reproduction number $({\mathbf{R}}_{0})$

The basic reproduction number can be determined using the method of next-generation matrix^33^. The next-generation matrix is the production of matrices $\mathrm{T}$ and $- {\Sigma }^{-1}$ where the matrix $\mathrm{T}$ represents the rate of infection transmission in $\mathrm{E}$ and $\mathrm{I}$ compartments and the matrix $\Sigma$ describes all other transfer across the compartments. The matrices $\mathrm{T}$ and $\Sigma$ are given as

$${\text{T}} = \left( {\begin{array}{*{20}l} 0 \hfill & {{\upbeta S}^{0} } \hfill \\ 0 \hfill & 0 \hfill \\ \end{array} } \right)\;{\text{and}}\; {\Sigma } = \left( {\begin{array}{*{20}c} { - \left( {{\upalpha } + {\upmu }} \right)} & 0 \\ {\upalpha } & { - \left( {{\upgamma } + {\updelta } + {\upmu }} \right)} \\ \end{array} } \right).$$

The next-generation matrix is

$$\mathrm{K}=\mathrm{T}\times \left(-{\Sigma }^{-1}\right)=\left(\begin{array}{cc}0&\upbeta {\mathrm{S}}^{0}\\ 0& 0\end{array}\right)\times \left(\begin{array}{cc}\frac{1}{(\mathrm{\upalpha }+\upmu )}& 0\\ \frac{\mathrm{\upalpha }}{(\mathrm{\upalpha }+\upmu )(\upgamma +\updelta +\upmu )}& \frac{1}{(\upgamma +\updelta +\upmu )}\end{array}\right) =\left(\begin{array}{cc}\frac{\upbeta {\mathrm{S}}^{0}\mathrm{\upalpha }}{(\mathrm{\upalpha }+\upmu )(\upgamma +\updelta +\upmu )}& \frac{\upbeta {\mathrm{S}}^{0}}{(\upgamma +\updelta +\upmu )}\\ 0& 0\end{array}\right).$$

The basic reproduction number is the Eigen-value of the largest magnitude of the next-generation matrix $(\mathrm{K})$. Hence the basic reproduction number is obtained as

$${\mathrm{R}}_{0}=\frac{\upbeta {\mathrm{S}}^{0}\mathrm{\upalpha }}{(\mathrm{\upalpha }+\upmu )(\upgamma +\updelta +\upmu )}=\frac{\mathrm{\upalpha \upbeta \upmu N}\left(\uprho +\upsigma +\upmu \right)}{(\mathrm{\upalpha }+\upmu )(\upgamma +\updelta +\upmu )\left(\left(\upeta +\upmu \right)\left(\uprho +\upsigma +\upmu \right)-\mathrm{\uprho \upeta }\right)}.$$

### Endemic equilibrium point $({\mathbf{X}}^{*})$

The endemic equilibrium point of the system (9)–(13) is discovered by applying $\mathrm{S}\ne {\mathrm{V}}_{1}\ne {\mathrm{V}}_{2}\ne \mathrm{E}\ne \mathrm{I}\ne 0$. Hence, the endemic equilibrium point is given by.

$${\mathrm{X}}^{*}=\left({\mathrm{S}}^{*}, {\mathrm{V}}_{1}^{*}, {\mathrm{V}}_{2}^{*}, {\mathrm{E}}^{*}, {\mathrm{I}}^{*}\right)$$ where

15 $$\left\{\begin{array}{l}{\mathrm{S}}^{*}=\frac{\left(\mathrm{\upalpha }+\upmu \right)\left(\upgamma +\updelta +\upmu \right)}{\mathrm{\upalpha \upbeta }}, \\ {\mathrm{V}}_{1}^{*}=\frac{\upeta \left(\mathrm{\upalpha }+\upmu \right)\left(\upgamma +\updelta +\upmu \right)}{\mathrm{\upalpha \upbeta }\left(\uprho +\updelta +\upmu \right)}, \\ {\mathrm{V}}_{2}^{*}=\frac{\mathrm{\sigma \upeta }\left(\mathrm{\upalpha }+\upmu \right)\left(\upgamma +\updelta +\upmu \right)}{\mathrm{\upalpha \upbeta }\left(\upomega +\upmu \right)\left(\uprho +\updelta +\upmu \right)}, \\ {\mathrm{E}}^{*}=\frac{\left({\mathrm{R}}_{0}-1\right)\left(\mathrm{\upalpha }+\upmu \right){\left(\upgamma +\updelta +\upmu \right)}^{2}\left(\left(\upeta +\upmu \right)\left(\uprho +\upsigma +\upmu \right)-\mathrm{\uprho \upeta }\right)}{\mathrm{\upalpha }\left(\left(\mathrm{\upalpha }+\upmu \right)\left(\upgamma +\updelta +\upmu \right)\right)\left(\uprho +\upsigma +\upmu \right)},\\ {\mathrm{I}}^{*}=\frac{\left({\mathrm{R}}_{0}-1\right)\left(\mathrm{\upalpha }+\upmu \right)(\upgamma +\updelta +\upmu )\left(\left(\upeta +\upmu \right)\left(\uprho +\upsigma +\upmu \right)-\mathrm{\uprho \upeta }\right)}{\left(\left(\mathrm{\upalpha }+\upmu \right)\left(\upgamma +\updelta +\upmu \right)\right)(\uprho +\upsigma +\upmu )}.\end{array}\right.$$

Equation (15) shows that if ${\mathrm{R}}_{0}>1$ then the endemic equilibrium ${\mathrm{X}}^{*}({\mathrm{S}}^{*}, {\mathrm{V}}_{1}^{*}, {\mathrm{V}}_{2}^{*}, {\mathrm{E}}^{*}, {\mathrm{I}}^{*})\in \mathrm{D}$.

### Stability analysis

To examining the stability of the equilibria of system (9)–(13), the following outcomes are proven:

#### Lemma 1

*The disease-free equilibrium of the model is locally asymptotically stable if*${\mathrm{R}}_{0}<1$* and unstable if*${\mathrm{R}}_{0}>1$.

#### ***Proof***

We consider the Jacobian of the system (9)–(13) which is given by

$$\mathrm{J}=\left(\begin{array}{c}\begin{array}{c}-(\upbeta I+\upeta +\upmu )\\ \upeta \\ 0\\ \upbeta I\\ 0\end{array}\end{array}\begin{array}{c}\begin{array}{c}\uprho \\ -(\uprho +\sigma +\upmu )\\ \sigma \\ 0\\ 0\end{array}\end{array}\begin{array}{c}\begin{array}{c}0\\ 0\\ -(\omega +\upmu )\\ 0\\ 0\end{array}\end{array}\begin{array}{cc}\begin{array}{c}\begin{array}{c}0\\ 0\\ 0\\ -(\upalpha +\upmu )\\ \upalpha \end{array}\end{array}& \begin{array}{c}\begin{array}{c}-\upbeta S\\ 0\\ 0\\ \upbeta S\\ -(\upgamma +\delta +\upmu )\end{array}\end{array}\end{array}\right)$$

which, at the infection-free equilibrium point, ${\mathrm{X}}^{0}$, reduces to

$$\mathrm{J}\left({\mathrm{X}}^{0}\right)=\left(\begin{array}{c}\begin{array}{c}-(\upeta +\upmu )\\ \upeta \\ 0\\ 0\\ 0\end{array}\end{array}\begin{array}{c}\begin{array}{c}\uprho \\ -(\uprho +\sigma +\upmu )\\ \sigma \\ 0\\ 0\end{array}\end{array}\begin{array}{c}\begin{array}{c}0\\ 0\\ -(\omega +\upmu )\\ 0\\ 0\end{array}\end{array}\begin{array}{cc}\begin{array}{c}\begin{array}{c}0\\ 0\\ 0\\ -(\upalpha +\upmu )\\ \upalpha \end{array}\end{array}& \begin{array}{c}\begin{array}{c}-\upbeta {\mathrm{S}}^{0}\\ 0\\ 0\\ \upbeta {\mathrm{S}}^{0}\\ -(\upgamma +\delta +\upmu )\end{array}\end{array}\end{array}\right).$$

Now we have to provide that all the eigenvalues of $\mathrm{J}\left({\mathrm{X}}^{0}\right)$ are negative. As the 3rd column indicates only the diagonal terms which form the one negative eigenvalue, $-(\upomega +\upmu )$ the other eigenvalues can be derived from the sub-matrix, ${\mathrm{J}}_{1}({\mathrm{X}}^{0})$ formed by excluding the 3rd row and column of $\mathrm{J}\left({\mathrm{X}}^{0}\right)$. Which gives

$${\mathrm{J}}_{1}({\mathrm{X}}^{0})=\left(\begin{array}{c}\begin{array}{c}-(\upeta +\upmu )\\ \upeta \\ 0\\ 0\end{array}\end{array}\begin{array}{c}\begin{array}{c}\uprho \\ -(\uprho +\sigma +\upmu )\\ 0\\ 0\end{array}\end{array}\begin{array}{cc}\begin{array}{c}\begin{array}{c}0\\ 0\\ -(\upalpha +\upmu )\\ \upalpha \end{array}\end{array}& \begin{array}{c}\begin{array}{c}-\upbeta {\mathrm{S}}^{0}\\ 0\\ \upbeta {\mathrm{S}}^{0}\\ -(\upgamma +\delta +\upmu )\end{array}\end{array}\end{array}\right).$$

This matrix can be written in block form as

$${\mathrm{J}}_{1}({\mathrm{X}}^{0})=\left(\begin{array}{cc}{\mathrm{A}}_{1}& {\mathrm{A}}_{2}\\ {\mathrm{A}}_{3}& {\mathrm{A}}_{4}\end{array}\right)$$

where, $${\mathrm{A}}_{1}=\left(\begin{array}{cc}-(\upeta +\upmu )&\uprho \\\upeta & -(\uprho +\upsigma +\upmu )\end{array}\right)$$, $${\mathrm{A}}_{2}=\left(\begin{array}{cc}0& -\upbeta {\mathrm{S}}^{0}\\ 0& 0\end{array}\right)$$, $${\mathrm{A}}_{3}=\left(\begin{array}{cc}0& 0\\ 0& 0\end{array}\right)$$ and $${\mathrm{A}}_{4}=\left(\begin{array}{cc}-(\mathrm{\upalpha }+\upmu )&\upbeta {\mathrm{S}}^{0}\\ \mathrm{\upalpha }& -(\upgamma +\updelta +\upmu )\end{array}\right).$$

The characteristic equation of the two-by-two block matrix ${\mathrm{J}}_{1}({\mathrm{X}}^{0})$ is

$$\mathrm{det}\left({\mathrm{A}}_{1}-\mathrm{\lambda I}\right)\mathrm{det}(\left({\mathrm{A}}_{4}-\mathrm{\lambda I}\right)-{\mathrm{A}}_{3}{\left({\mathrm{A}}_{1}-\mathrm{\lambda I}\right)}^{-1}{\mathrm{A}}_{2})=0,$$

Since $${\mathrm{A}}_{3}=\left(\begin{array}{cc}0& 0\\ 0& 0\end{array}\right)$$ this reduces to

$$\mathrm{det}\left({\mathrm{A}}_{1}-\mathrm{\lambda I}\right)\mathrm{det}\left({\mathrm{A}}_{4}-\mathrm{\lambda I}\right)=0.$$

Now we can apply the Routh-Hurwitz criteria for stability to matrices ${\mathrm{A}}_{1}$ and ${\mathrm{A}}_{4}$ directly and independently. We require that the trace is negative and the determinant is positive for each matrix.

Now for ${\mathrm{A}}_{1}$ matrix

$$\mathrm{trace}\left({\mathrm{A}}_{1}\right)=-(\upeta +\upmu )-(\uprho +\upsigma +\upmu )<0,$$

and

$$\mathrm{det}\left({\mathrm{A}}_{1}\right)=\left(\upeta +\upmu \right)\left(\uprho +\upsigma +\upmu \right)-\mathrm{\uprho \upeta }=\upeta \left(\upsigma +\upmu \right)+\upmu \left(\uprho +\upsigma +\upmu \right)>0.$$

Again for ${\mathrm{A}}_{4}$ matrix

$$\mathrm{trace}\left({\mathrm{A}}_{4}\right)=-(\mathrm{\upalpha }+\upmu )-(\upgamma +\updelta +\upmu )<0,$$

and

$$\mathrm{det}\left({\mathrm{A}}_{4}\right)=\left(\mathrm{\upalpha }+\upmu \right)\left(\upgamma +\updelta +\upmu \right)-\mathrm{\upalpha \upbeta }{\mathrm{S}}^{0}=1-\frac{\mathrm{\upalpha \upbeta }{\mathrm{S}}^{0}}{\left(\mathrm{\upalpha }+\upmu \right)\left(\upgamma +\updelta +\upmu \right)}>0,$$

which we can rewrite as

$${\mathrm{R}}_{0}<1.$$

Hence, the disease-free equilibrium ${\mathrm{X}}^{0}$ is locally asymptotically stable for ${\mathrm{R}}_{0}<1$. If either ${\mathrm{R}}_{0}>1$, at least one of the roots of the characteristic equation has a positive real part and ${\mathrm{X}}^{0}$ is unstable.

#### Lemma 2

*The endemic equilibrium*${\mathrm{X}}^{*}$* is locally asymptotically stable if *${\mathrm{R}}_{0}>1$.

#### ***Proof***

We consider the Jacobian of the system (9)–(13) at ${\mathrm{X}}^{*}=({\mathrm{S}}^{*}, {\mathrm{V}}_{1}^{*}, {\mathrm{V}}_{2}^{*}, {\mathrm{E}}^{*}, {\mathrm{I}}^{*})$ which is given by

$$\mathrm{J}({\mathrm{X}}^{*})=\left(\begin{array}{c}\begin{array}{c}-(\upbeta {\mathrm{I}}^{*}+\upeta +\upmu )\\ \upeta \\ 0\\ \upbeta {\mathrm{I}}^{*}\\ 0\end{array}\end{array}\begin{array}{c}\begin{array}{c}\uprho \\ -(\uprho +\sigma +\upmu )\\ \sigma \\ 0\\ 0\end{array}\end{array}\begin{array}{c}\begin{array}{c}0\\ 0\\ -(\omega +\upmu )\\ 0\\ 0\end{array}\end{array}\begin{array}{cc}\begin{array}{c}\begin{array}{c}0\\ 0\\ 0\\ -(\upalpha +\upmu )\\ \upalpha \end{array}\end{array}& \begin{array}{c}\begin{array}{c}-\upbeta {\mathrm{S}}^{*}\\ 0\\ 0\\ \upbeta {\mathrm{S}}^{*}\\ -(\upgamma +\delta +\upmu )\end{array}\end{array}\end{array}\right).$$

The 3rd column indicates only the diagonal terms which form the one negative eigenvalues,$-(\upomega +\upmu )$, the other eigenvalues can be derived from the sub-matrix, ${\mathrm{J}}_{1}({\mathrm{X}}^{*})$ formed by excluding the 3rd rows and columns of $\mathrm{J}({\mathrm{X}}^{*})$. Which gives

$${\mathrm{J}}_{1}\left({\mathrm{X}}^{*}\right)=\left(\begin{array}{c}\begin{array}{c}-(\upbeta {\mathrm{I}}^{*}+\upeta +\upmu )\\ \upeta \\ \upbeta {\mathrm{I}}^{*}\\ 0\end{array}\end{array}\begin{array}{c}\begin{array}{c}\uprho \\ -(\uprho +\sigma +\upmu )\\ 0\\ 0\end{array}\end{array}\begin{array}{cc}\begin{array}{c}\begin{array}{c}0\\ 0\\ -(\upalpha +\upmu )\\ \upalpha \end{array}\end{array}& \begin{array}{c}\begin{array}{c}-\upbeta {\mathrm{S}}^{*}\\ 0\\ \upbeta {\mathrm{S}}^{*}\\ -(\upgamma +\delta +\upmu )\end{array}\end{array}\end{array}\right).$$

The characteristics equation of ${\mathrm{J}}_{1}\left({\mathrm{X}}^{*}\right)$ is defined as,

$$\left|{\mathrm{J}}_{1}\left({\mathrm{X}}^{*}\right)-\mathrm{\lambda I}\right|=0,$$

16 $$\begin{aligned}& \Rightarrow \left|\begin{array}{c}\begin{array}{c}-(\beta {\mathrm{I}}^{*}+\eta +\mu +\lambda )\\ \eta \\ \beta {\mathrm{I}}^{*}\\ 0\end{array}\end{array}\begin{array}{c}\begin{array}{c}\rho \\ -(\rho +\sigma +\mu +\lambda )\\ 0\\ 0\end{array}\end{array}\begin{array}{cc}\begin{array}{c}\begin{array}{c}0\\ 0\\ -(\alpha +\mu +\lambda )\\ \alpha \end{array}\end{array}& \begin{array}{c}\begin{array}{c}-\beta {\mathrm{S}}^{*}\\ 0\\ \beta {\mathrm{S}}^{*}\\ -(\gamma +\delta +\mu +\lambda )\end{array}\end{array}\end{array}\right|=0, \\ & \Rightarrow -\left(\upbeta {\mathrm{I}}^{*}+\upeta +\upmu +\uplambda \right)\left|\begin{array}{ccc}-\left(\uprho +\upsigma +\upmu +\uplambda \right)& 0& 0\\ 0& -\left(\mathrm{\alpha }+\upmu +\uplambda \right)&\upbeta {\mathrm{S}}^{*}\\ 0& \mathrm{\alpha }& -\left(\upgamma +\updelta +\upmu +\uplambda \right)\end{array}\right| \\ & \quad -\uprho \left|\begin{array}{ccc}\upeta & 0& 0\\\upbeta {\mathrm{I}}^{*}& -\left(\mathrm{\alpha }+\upmu +\uplambda \right)&\upbeta {\mathrm{S}}^{*}\\ 0& \mathrm{\alpha }& -\left(\upgamma +\updelta +\upmu +\uplambda \right)\end{array}\right| \\ & \quad + \left(\upbeta {\mathrm{S}}^{*}\right)\left|\begin{array}{ccc}\upeta & -\left(\uprho +\upsigma +\upmu +\uplambda \right)& 0\\\upbeta {\mathrm{I}}^{*}& 0& -\left(\mathrm{\alpha }+\upmu +\uplambda \right)\\ 0& 0& \mathrm{\alpha }\end{array}\right|= 0, \\ & \Rightarrow {\uplambda }^{4}+{\mathrm{a}}_{3}{\uplambda }^{3}+{\mathrm{a}}_{2}{\uplambda }^{2}+{\mathrm{a}}_{1}\uplambda +{\mathrm{a}}_{0}=0. \end{aligned}$$

where

$${\mathrm{a}}_{3}=\left(\upbeta {\mathrm{I}}^{*}+\upeta +\upmu \right)+\left(\uprho +\upsigma +\upmu \right)+\left(\mathrm{\upalpha }+\upmu \right)+\left(\upgamma +\updelta +\upmu \right),$$

$$\begin{aligned}{\mathrm{a}}_{2}&=\left(\upbeta {\mathrm{I}}^{*}+\upeta +\upmu \right)\left(\uprho +\upsigma +\upmu \right)+\left(\upbeta {\mathrm{I}}^{*}+\upeta +\upmu \right)\left(\mathrm{\upalpha }+\upmu \right)+\left(\uprho +\upsigma +\upmu \right)\left(\mathrm{\upalpha }+\upmu \right) \\ & \quad +\left(\upbeta {\mathrm{I}}^{*}+\upeta +\upmu \right)\left(\upgamma +\updelta +\upmu \right)+\left(\uprho +\upsigma +\upmu \right)\left(\upgamma +\updelta +\upmu \right)+\left(\mathrm{\upalpha }+\upmu \right)\left(\upgamma +\updelta +\upmu \right) \\ & \quad -\mathrm{\upeta \uprho }-\mathrm{\upalpha \upbeta }{\mathrm{S}}^{*}, \\ & =\left(\upbeta {\mathrm{I}}^{*}+\upeta +\upmu \right)\left(\uprho +\upsigma +\upmu \right)+\left(\upbeta {\mathrm{I}}^{*}+\upeta +\upmu \right)\left(\mathrm{\upalpha }+\upmu \right)+\left(\uprho +\upsigma +\upmu \right)\left(\mathrm{\upalpha }+\upmu \right) \\ & \quad +\left(\upbeta {\mathrm{I}}^{*}+\upeta +\upmu \right)\left(\upgamma +\updelta +\upmu \right)+\left(\uprho +\upsigma +\upmu \right)\left(\upgamma +\updelta +\upmu \right)+\left(\mathrm{\upalpha }+\upmu \right)\left(\upgamma +\updelta +\upmu \right) \\ &\quad -\mathrm{\upeta \uprho }-\left(\mathrm{\upalpha }+\upmu \right)\left(\upgamma +\updelta +\upmu \right), \end{aligned}$$

$$\begin{aligned}\Rightarrow {\mathrm{a}}_{2}& =\uprho \left(\upbeta {\mathrm{I}}^{*}+\upmu \right)+\left(\upbeta {\mathrm{I}}^{*}+\upeta +\upmu \right)\left(\sigma +\upmu \right)+\left(\upbeta {\mathrm{I}}^{*}+\upeta +\upmu \right)\left(\mathrm{\upalpha }+\upmu \right)+\left(\uprho +\upsigma +\upmu \right)\left(\mathrm{\upalpha }+\upmu \right) \\ & \quad +\left(\upbeta {\mathrm{I}}^{*}+\upeta +\upmu \right)\left(\upgamma +\updelta +\upmu \right)+\left(\uprho +\upsigma +\upmu \right)\left(\upgamma +\updelta +\upmu \right), \end{aligned}$$

$$\begin{aligned}{\mathrm{a}}_{1}&=\left(\upbeta {\mathrm{I}}^{*}+\upeta +\upmu \right)\left(\uprho +\upsigma +\upmu \right)\left(\mathrm{\upalpha }+\upmu \right)+\left(\upbeta {\mathrm{I}}^{*}+\upeta +\upmu \right)\left(\uprho +\upsigma +\upmu \right)\left(\upgamma +\updelta +\upmu \right) \\ & \quad +\left(\upbeta {\mathrm{I}}^{*}+\upeta +\upmu \right)\left(\mathrm{\upalpha }+\upmu \right)\left(\upgamma +\updelta +\upmu \right)+\left(\uprho +\upsigma +\upmu \right)\left(\mathrm{\upalpha }+\upmu \right)\left(\upgamma +\updelta +\upmu \right) \\ & \quad -\left(\mathrm{\upalpha }+\upmu \right)\mathrm{\upeta \uprho }-\left(\upgamma +\updelta +\upmu \right)\mathrm{\upeta \uprho }-\mathrm{\upalpha \upbeta }\left(\upbeta {\mathrm{I}}^{*}+\upeta +\upmu \right){\mathrm{S}}^{*}-\mathrm{\upalpha \upbeta }\left(\uprho +\upsigma +\upmu \right){\mathrm{S}}^{*}+\mathrm{\upalpha }{\upbeta }^{2}{\mathrm{S}}^{*}{\mathrm{I}}^{*}, \end{aligned}$$

On simplify

$$\begin{aligned}{\mathrm{a}}_{1}&=\uprho \left(\upbeta {\mathrm{I}}^{*}+\upmu \right)\left(\mathrm{\upalpha }+\upmu \right)+\left(\upbeta {\mathrm{I}}^{*}+\upeta +\upmu \right)\left(\upsigma +\upmu \right)\left(\mathrm{\upalpha }+\upmu \right)+\uprho \left(\upbeta {\mathrm{I}}^{*}+\upmu \right)\left(\upgamma +\updelta +\upmu \right)\\ & \quad +\left(\upbeta {\mathrm{I}}^{*}+\upeta +\upmu \right)\left(\upsigma +\upmu \right)\left(\upgamma +\updelta +\upmu \right)+\upbeta \left(\mathrm{\upalpha }+\upmu \right)\left(\upgamma +\updelta +\upmu \right){\mathrm{I}}^{*}, \end{aligned}$$

and

17 $$\begin{aligned}{\mathrm{a}}_{0} &= \left(\upbeta {\mathrm{I}}^{*}+\upeta +\upmu \right)\left(\uprho +\upsigma +\upmu \right)\left(\mathrm{\alpha }+\upmu \right)\left(\upgamma +\updelta +\upmu \right)-\left(\mathrm{\alpha }+\upmu \right)\left(\upgamma +\updelta +\upmu \right)\mathrm{\eta \rho } \\ & \quad +\mathrm{\alpha \beta \eta \rho }{\mathrm{S}}^{*}+\mathrm{\alpha }{\upbeta }^{2}\left(\uprho +\upsigma +\upmu \right){\mathrm{S}}^{*}{\mathrm{I}}^{*}-\mathrm{\alpha \beta }\left(\upbeta {\mathrm{I}}^{*}+\upeta +\upmu \right)\left(\uprho +\upsigma +\upmu \right){\mathrm{S}}^{*}, \\ &=\left(\upbeta {\mathrm{I}}^{*}+\upeta +\upmu \right)\left(\uprho +\upsigma +\upmu \right)\left(\mathrm{\alpha }+\upmu \right)\left(\upgamma +\updelta +\upmu \right)-\left(\mathrm{\alpha }+\upmu \right)\left(\upgamma +\updelta +\upmu \right)\mathrm{\eta \rho } \\ & \quad +\left(\mathrm{\alpha }+\upmu \right)\left(\upgamma +\updelta +\upmu \right)\mathrm{\eta \rho }+\upbeta \left(\mathrm{\alpha }+\upmu \right)\left(\upgamma +\updelta +\upmu \right)\left(\uprho +\upsigma +\upmu \right){\mathrm{I}}^{*} \\ & \quad -\left(\upbeta {\mathrm{I}}^{*}+\upeta +\upmu \right)\left(\uprho +\upsigma +\upmu \right)\left(\mathrm{\alpha }+\upmu \right)\left(\upgamma +\updelta +\upmu \right), \\ & \Rightarrow {\mathrm{a}}_{0}=\upbeta \left(\mathrm{\alpha }+\upmu \right)\left(\upgamma +\updelta +\upmu \right)\left(\uprho +\upsigma +\upmu \right){\mathrm{I}}^{*}.\end{aligned}$$

From (17) it is easy to verify that

${\mathrm{a}}_{3}>0, \, {\mathrm{a}}_{2}>0, \, {\mathrm{a}}_{1}>0$ and ${\mathrm{a}}_{0}>0$ if ${\mathrm{I}}^{*}>0$. From (15) it is also clear that ${\mathrm{I}}^{*}$ is positive if ${\mathrm{R}}_{0}>1$.

Hence, by the Routh–Hurwitz stability criterion, the endemic equilibrium point ${\mathrm{X}}^{*}$ is locally asymptotically stable for ${\mathrm{R}}_{0}>1$.

**Figure 2:**
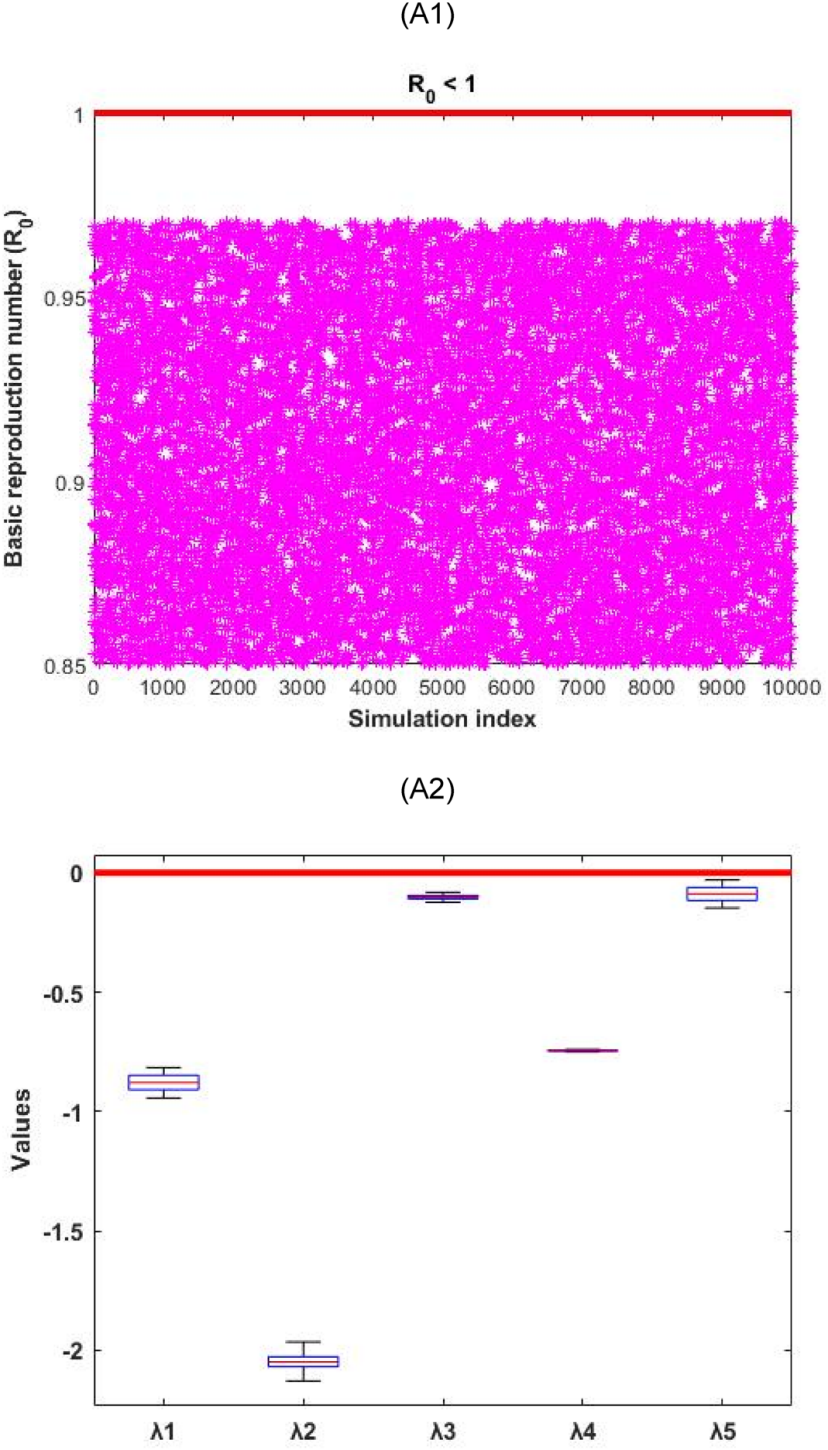
Numerical simulation for the infection-free equilibrium stability conditions and the real part distribution of the eigenvalues $({\uplambda }_{1}, \, {\uplambda }_{2}, \, {\uplambda }_{3}, \, {\uplambda }_{4}, \, {\uplambda }_{5})$. (A1) depict that ${\mathrm{R}}_{0}<1$ always hold, and (A2) represents the related distribution of the real part of the eigenvalues for the disease-free conditions.

**Figure 3:**
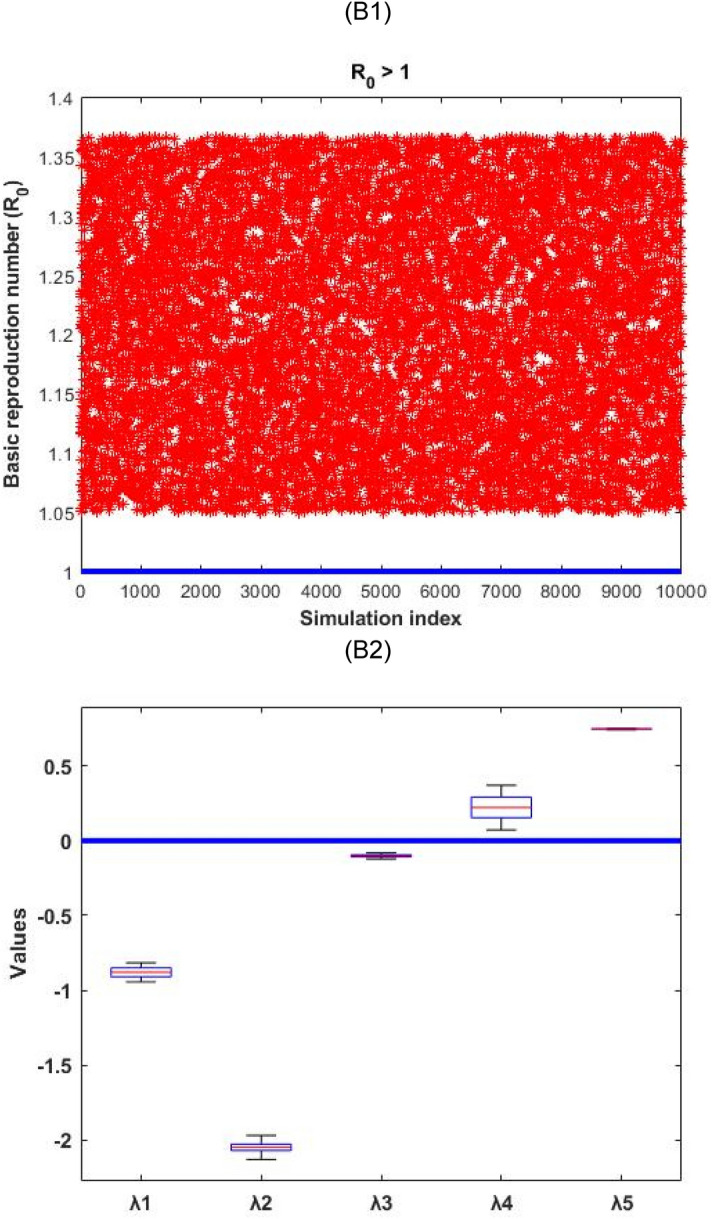
Numerical simulation for the endemic equilibrium stability conditions and the real part distribution of the eigenvalues $({\uplambda }_{1}, {\uplambda }_{2}, {\uplambda }_{3}, {\uplambda }_{4}, {\uplambda }_{5})$. (B1) depicts that ${\mathrm{R}}_{0}>1$ always hold, and (B2) represents the related distribution of the real part of the eigenvalues for the endemic conditions.

In order to verify the nature of the disease-free and endemic equilibrium analysis, we used Monte Carlo simulation^34^ to prove the conditions by calculating the real part of the eigenvalues of the Jacobian matrix of disease-free and endemic equilibriums. The simulation outcomes are presented in Figs. 2 and 3.

Figure 2 represented that the disease-free equilibrium is locally stable as all the five eigenvalues (real part) are negative $(\mathrm{i}.\mathrm{e}. \, {\uplambda }_{1}, \, {\uplambda }_{2}, \, {\uplambda }_{3}, \, {\uplambda }_{4}, \, \mathrm{ and } \, {\uplambda }_{5}<0)$. Whilst Fig. 3 represented that the endemic equilibrium is unstable as two eigenvalues (real part) is positive $(\mathrm{i}.\mathrm{e}. \, {\uplambda }_{4}, \, {\uplambda }_{5}>0)$.

### Parameters estimation

**Figure 4:**
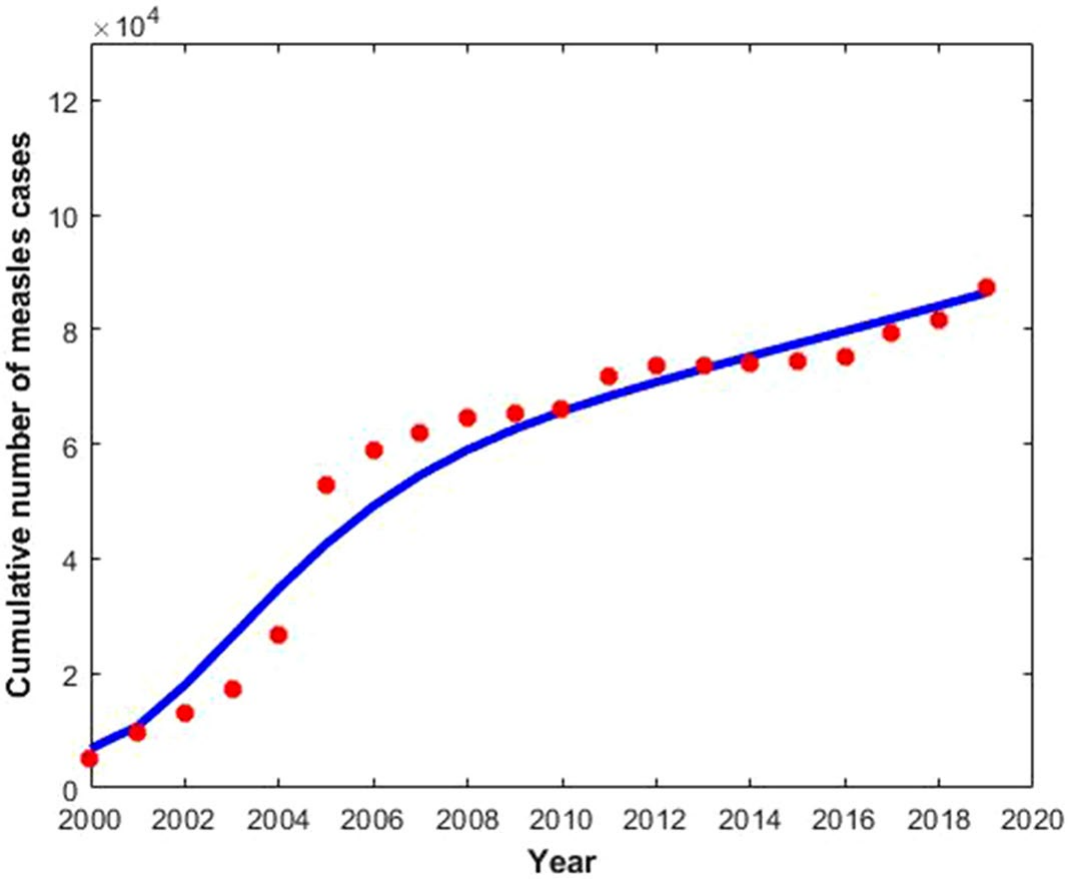
Cumulative number of confirmed measles cases from 2000 to 2019 (red dot) and the corresponding model best fit (solid blue curve) in Bangladesh.

We estimated the measles model parameters from fitting different combinations of parameters in Eqs. (2)–(7) to the actual number of measles cases in Bangladesh from 2000 to 2019^35^. In order to parameterise measles model (2)–(7), we obtained some of the parameter values from the literature (see Table 1), rest of the parameters were estimated from data fitting (see Fig. 4). The estimation of parameters was carried out using the least-squared method, which minimises summation of the square errors given by $\sum {\left(\mathrm{M}\left(\mathrm{t},\mathrm{p}\right)-{\mathrm{N}}_{\mathrm{actual}}\right)}^{2}$ subject to the measles model (2)–(7), where ${\mathrm{N}}_{\mathrm{actual}}$ is the actual reported measles data, and $\mathrm{M}\left(\mathrm{t}, \, \mathrm{p}\right)$ denotes the solution of the model corresponding to the number of measles cases over time $\mathrm{t}$ with the set of estimated parameters, denoted by $\mathrm{p}$.

**Table 1:** Depiction and estimation of the measles model (2)–(7) parameters.

Parameters	Description	Estimated value	References
$\mathrm{N}$	Total population in Bangladesh	163,046,161	^40^
$\upmu$	Per-capita death rate	$\frac{1}{70}$ per year	^41^
$\upbeta$	Transmission rate	$7.45\times {10}^{-7}$	Fitted
$\upeta$	First dose of vaccine rate	0.94	^14^
$\uprho$	Progression rate from ${\mathrm{V}}_{1}$ to $\mathrm{S}$	0.6	^32^
$\upsigma$	Second dose of vaccine rate	0.93	^14^
$\upomega$	Recovery rate due to the second dose of vaccine	0.8	^32^
$\mathrm{\upalpha }$	Progression rate from $\mathrm{E}$ to $\mathrm{I}$	0.018	Fitted
$\updelta$	Measles related death rate	0.125	^32^
$\upgamma$	Natural recovery rate	0.6	^32^

### Estimation of basic reproduction number $({\mathbf{R}}_{0})$

The basic reproduction number is well-defined as the estimated number of secondary cases produced by a single infectious case presented into an exclusively susceptible population. The disease can spread in a population only if the basic reproduction number is greater than one. Our main objective here is to calculate the basic reproduction number $({\mathrm{R}}_{0})$ of the measles model (2)–(7). Using the values $\mathrm{\upalpha }=0.018,\upbeta =7.45\times {10}^{-7},\mathrm{ N}=\mathrm{163,046,161},\upmu =\frac{1}{70},\uprho =0.6,$ $\upsigma =0.93$,

$\upgamma =0.6, \, \updelta =0.125, \, \upeta =0.94$, and substituting it into the basic reproduction number $({\mathrm{R}}_{0})$ expression result in:

$${\mathrm{R}}_{0}=\frac{\mathrm{\upalpha \upbeta \upmu N}\left(\uprho +\upsigma +\upmu \right)}{(\mathrm{\upalpha }+\upmu )(\upgamma +\updelta +\upmu )\left(\left(\upeta +\upmu \right)\left(\uprho +\upsigma +\upmu \right)-\mathrm{\uprho \upeta }\right)},$$

$$=\frac{0.018\times 7.45\times {10}^{-7}\times \frac{1}{70}\times 163046161\times (0.6+0.93+\frac{1}{70})}{(0.018+\frac{1}{70})\times (0.6+0.125+\frac{1}{70})\left(\left(0.94+\frac{1}{70}\right)\times \left(0.6+0.93+\frac{1}{70}\right)-0.6\times 0.94\right)},$$

$$\approx 1.44.$$

Hence, the basic reproduction number, ${\mathrm{R}}_{0}$ is approximately 1.44. It indicates that a single infected individual can spread the measles disease to 1 or 2 susceptible individuals.

### Sensitivity analysis

**Figure 5:**
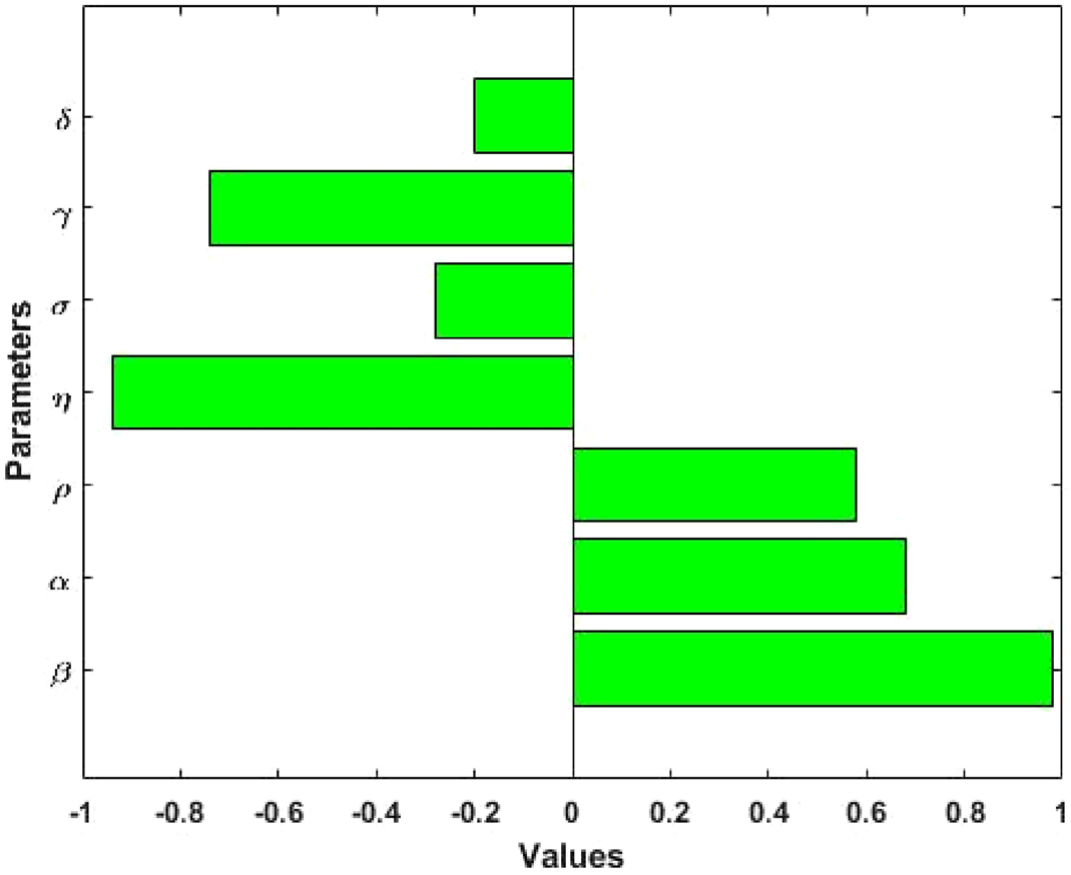
Correlation between basic reproduction number $({\mathrm{R}}_{0})$ and the model parameters $\upbeta , \, \mathrm{ \upalpha }, \, \uprho , \, \upsigma , \, \upgamma , \, \mathrm{ \upeta and \delta }$.

**Figure 6:**
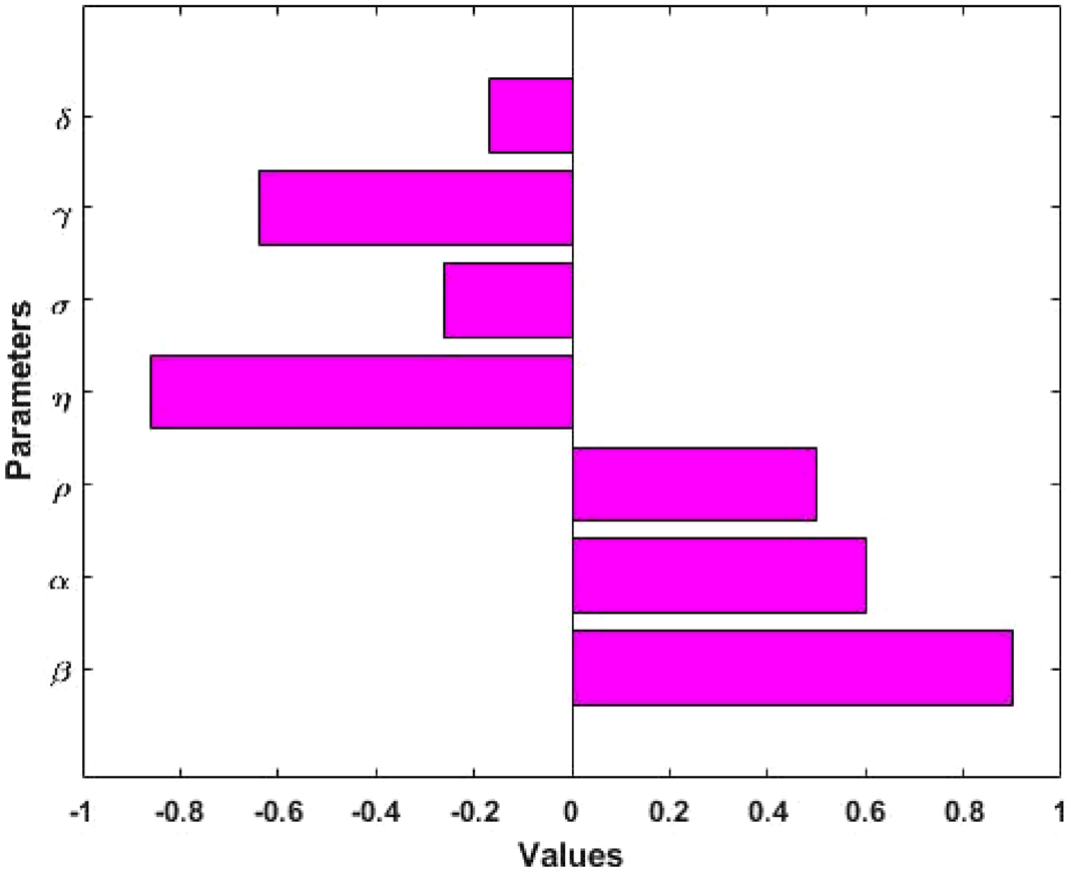
Correlation between measles prevalence $({\mathrm{I}}^{*})$ and the model parameters $\upbeta , \, \mathrm{ \upalpha }, \, \uprho , \, \upsigma , \, \upgamma , \, \mathrm{ \upeta and \delta }$.

We perform the sensitivity of the model basic reproduction number $({\mathrm{R}}_{0})$ and measles prevalence $({\mathrm{I}}^{*})$ to the model parameters using the Latin Hypercube Sampling (LHS) method with 10,000 runs per simulation. The LHS is a Monte Carlo stratified sampling technique that permits us to concurrently achieve an unbiased assessment of the model output for a particular set of input parameter values. We estimated the Partial Rank Correlation Coefficients (PRCCs): a global sensitivity analysis method using LHS of crucial output variables. We allowed a uniform distribution from 0 to 4 times the baseline value for each input parameter to explore the relationship between model output variable and parameters. The PRCCs for the basic reproduction number and measles prevalence in Figs. 5 and 6 have been produced using the expressions ${\mathrm{R}}_{0}$ and ${\mathrm{I}}^{*}$. Results show that parameters transmission rate $(\upbeta )$ and progression rates $(\mathrm{\upalpha and \uprho })$ have a positive correlation with the model outcomes ${\mathrm{R}}_{0}$ and ${\mathrm{I}}^{*}$, which means that decreasing these parameters values will reduce the prevalence of measles. On the other hand, parameters $\upeta , \, \upsigma , \, \upgamma$ and $\updelta$ have a negative correlation with the model outcomes ${\mathrm{R}}_{0}$ and ${\mathrm{I}}^{*}$, which indicates that increasing these parameters will decrease the prevalence of measles.

From the explicit formula for basic reproduction number ${\mathrm{R}}_{0}$, the analytical expression for the sensitivity indices can be derived applying the method in^36–39^ to each of the parameters, e.g.

$${\upgamma }_{{\mathrm{R}}_{0}}^{\upbeta }=\frac{\partial {\mathrm{R}}_{0}}{\partial\upbeta } \times \frac{\upbeta }{{\mathrm{R}}_{0}}.$$

Using the parameter values in Table 1, we have the following values and the nature of their signs in Table 2.

**Table 2:** Sensitivity indices of ${\mathrm{R}}_{0}$ to parameters for the model (2)–(7).

Parameter	Description	Sensitivity index $({\mathrm{R}}_{0})$
$\upbeta$	Transmission rate	+ 1.000
$\mathrm{\upalpha }$	Progression rate from $\mathrm{E}$ to $\mathrm{I}$	+ 0.443
$\uprho$	Progression rate from ${\mathrm{V}}_{1}$ to $\mathrm{S}$	+ 0.373
$\upeta$	First dose of vaccine rate	− 0.976
$\upsigma$	Second dose of vaccine rate	− 0.373
$\upgamma$	Natural recovery rate	− 0.812
$\updelta$	Measles related death rate	− 0.169

In the sensitivity indices of ${\mathrm{R}}_{0}$ the most sensitive parameter is the transmission rate $(\upbeta )$ of measles. Another significant parameter is the first dose of vaccination rate $(\upeta )$. The least sensitive parameter is the measles related death rate $(\updelta )$. Hence, increasing (or decreasing) the transmission rate $(\upbeta )$ of measles by 100% increases (or decreases) the basic reproduction number ${\mathrm{R}}_{0}$ by 100%. Similarly, increasing (or decreasing) the measles-related death rate $(\updelta )$ by 100% decreases (or increases) ${\mathrm{R}}_{0}$ by 16.9%.

### Numerical simulation

In this section, we carry out detailed numerical simulations to support the analytic results and to assess the impact of model parameters, including progression rate, transmission rate and double dose vaccination. For illustration, we have chosen baseline parameter values consistent with measles infection and transmission (see Table 1). We found two equilibrium points following the analytical results: the disease-free equilibrium $({\mathrm{X}}^{0})$ and an endemic equilibrium ${(\mathrm{X}}^{*})$. We used different initial conditions for the exposed and infected population and found that if the basic reproduction number less than one $(\mathrm{i}.\mathrm{e}.\, {\mathrm{R}}_{0}<1)$, then the disease-free equilibrium is locally asymptotically stable. Furthermore, if ${\mathrm{R}}_{0}>1$ then measles persists in the population.

**Figure 7:**
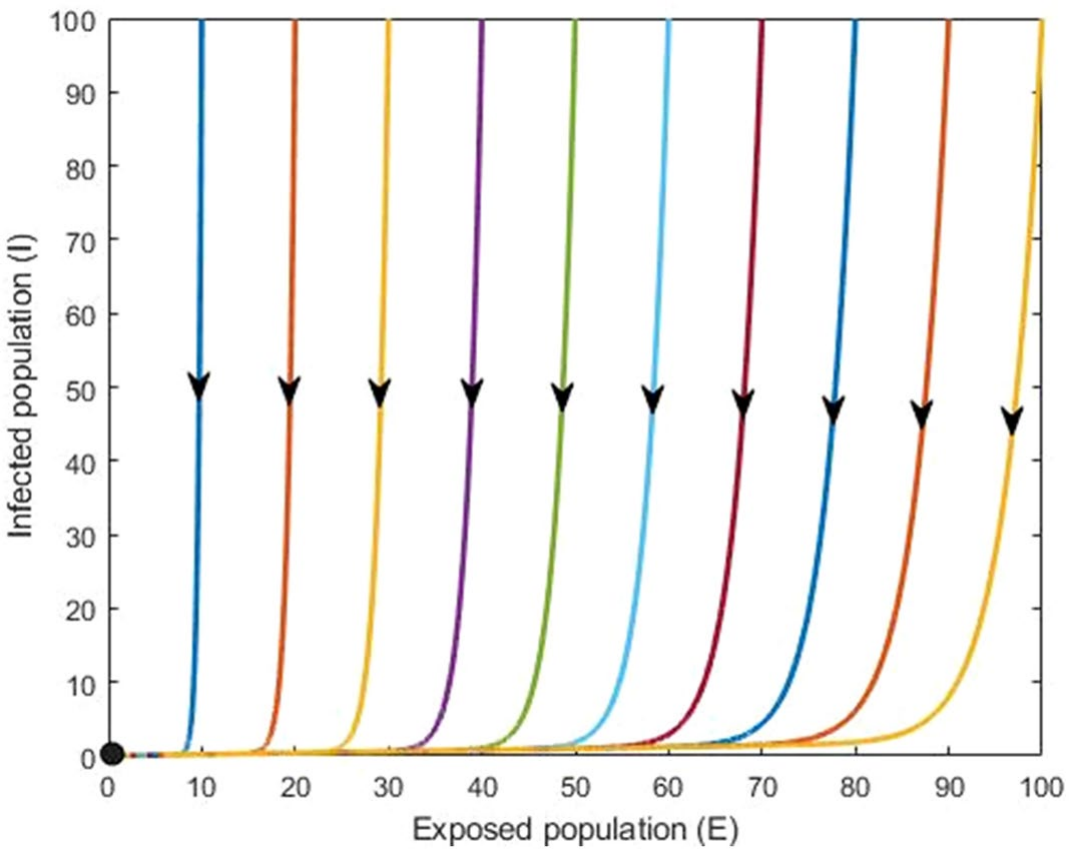
Infection-free equilibrium: ${\mathrm{R}}_{0}<1$. In this case measles disease dies out (black dot). All parameter values assume their baseline values given in Table 1.

**Figure 8:**
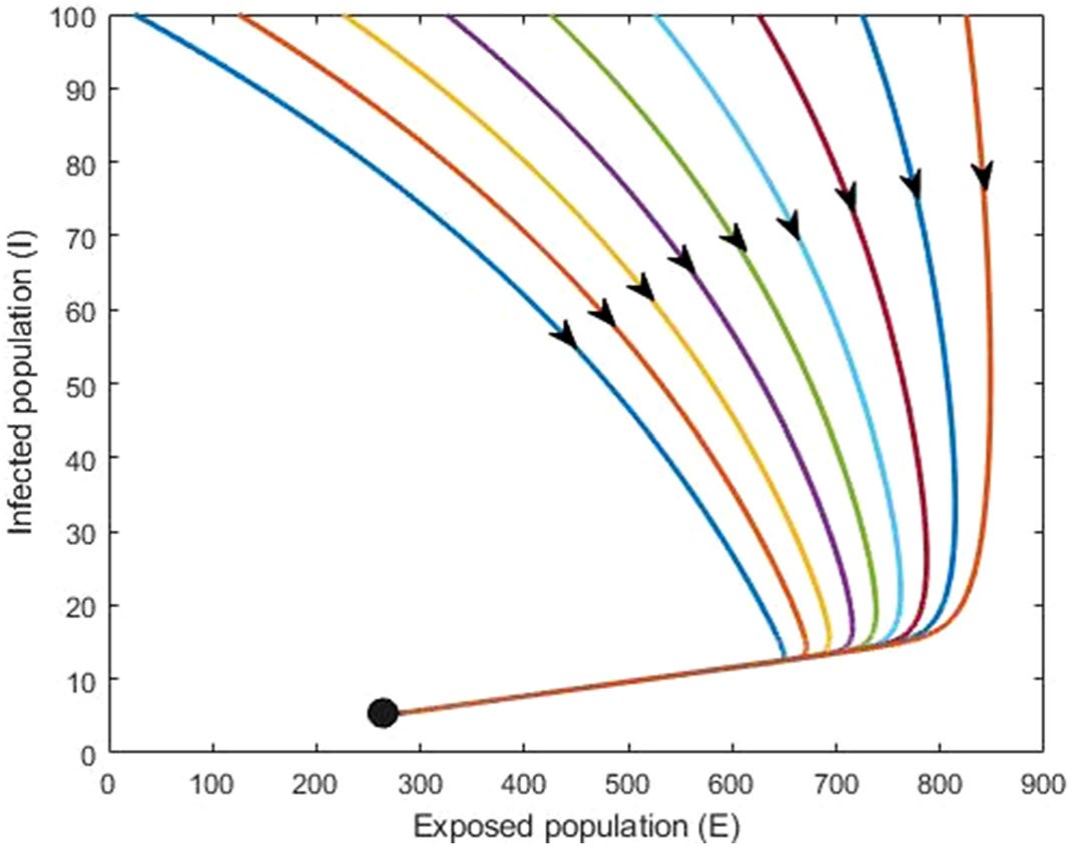
Endemic equilibrium: ${\mathrm{R}}_{0}>1$. In this case measles disease persist in the population (black dot). All parameter values assume their baseline values given in Table 1.

Figure 7 illustrates the stability of the disease-free equilibrium $(\mathrm{i}.\mathrm{e}.\,\mathrm{ when } {\mathrm{R}}_{0}<1)$ by depicting system trajectories through the $\mathrm{E vs} \mathrm{I}$ plane originating from different initial conditions. In this case, measles disease dies out. Figure 8 shows the stability of the endemic equilibrium $(\mathrm{i}.\mathrm{e}.\mathrm{ when } {\mathrm{R}}_{0}>1)$, and in this case, measles disease persists in the population.

**Figure 9:**
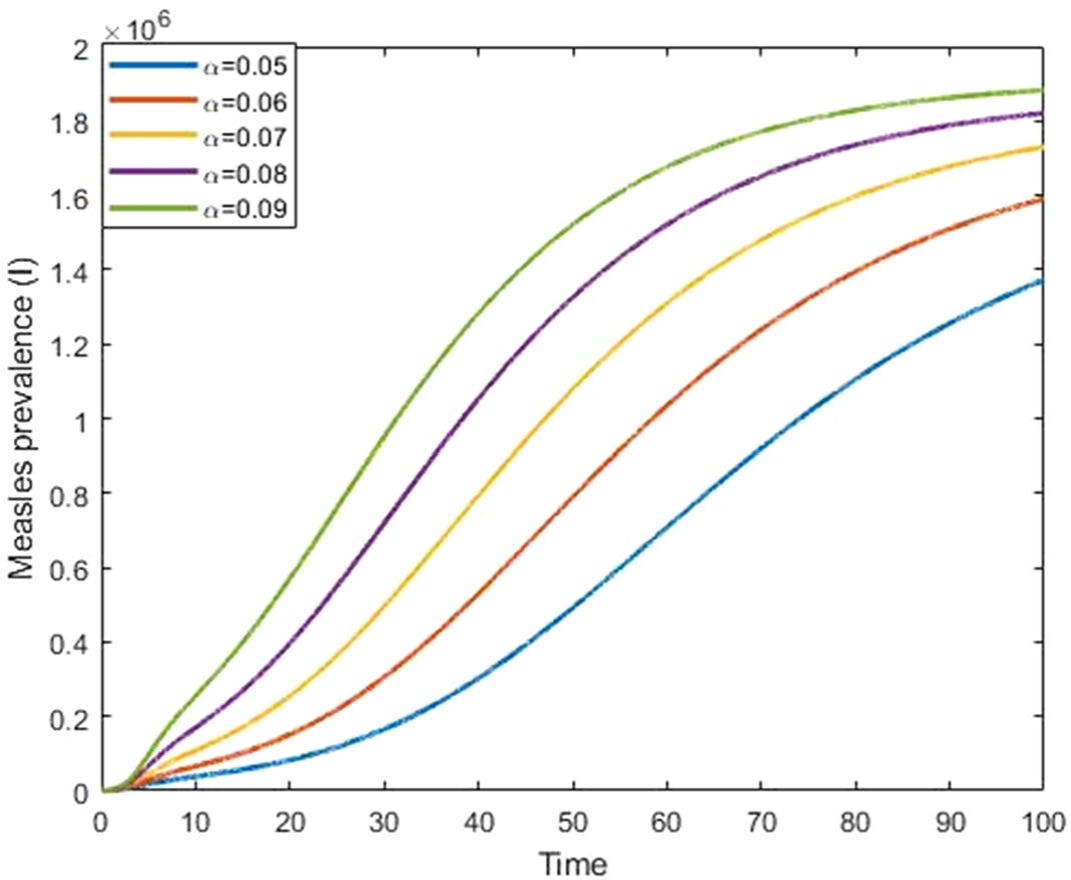
Impact of progression rate $(\mathrm{\upalpha })$ on measles prevalence.

**Figure 10:**
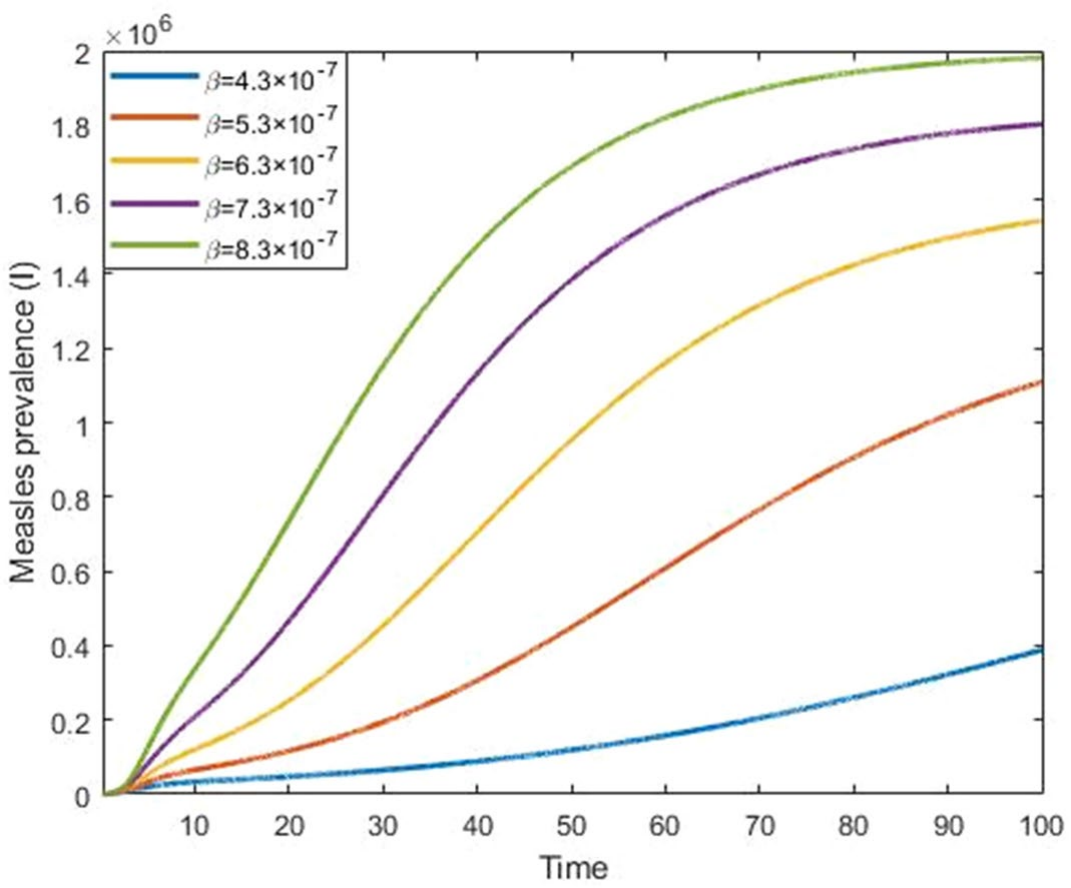
Impact of transmission rate $(\upbeta )$ on measles prevalence.

**Figure 11:**
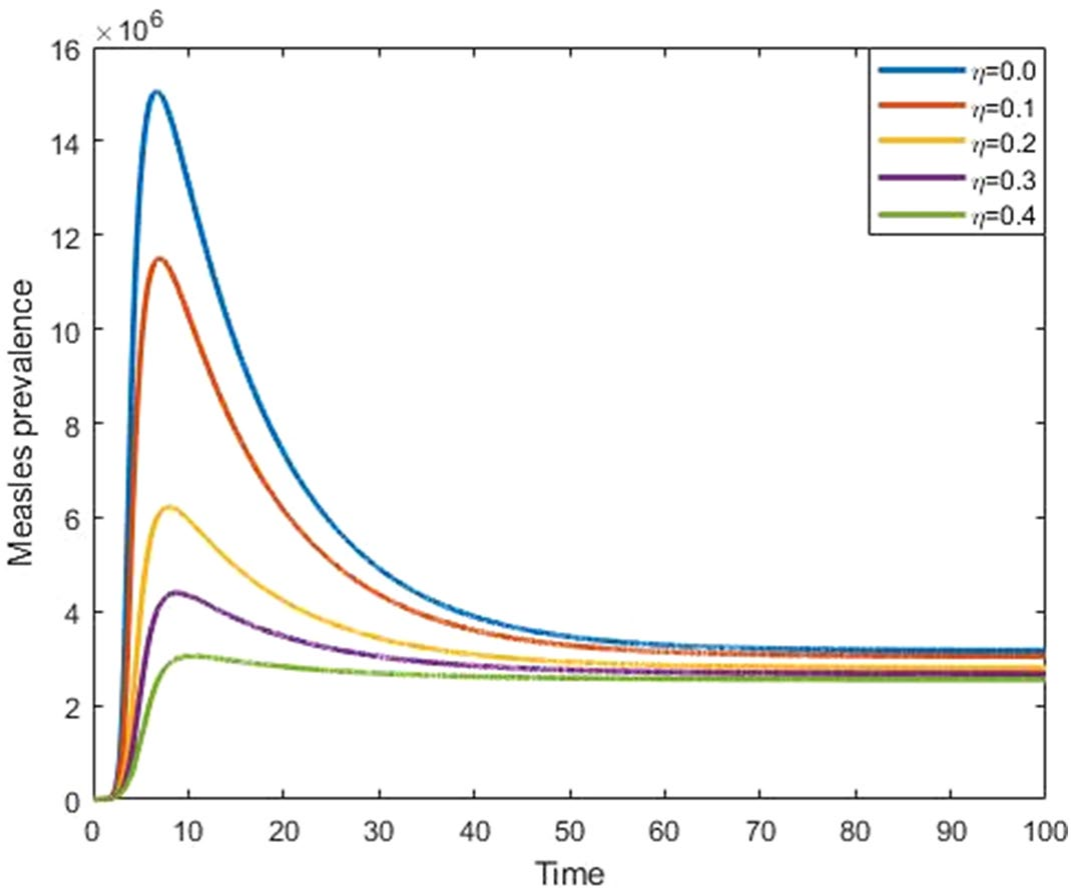
Impact of first dose vaccine $(\upeta )$ on measles prevalence.

**Figure 12:**
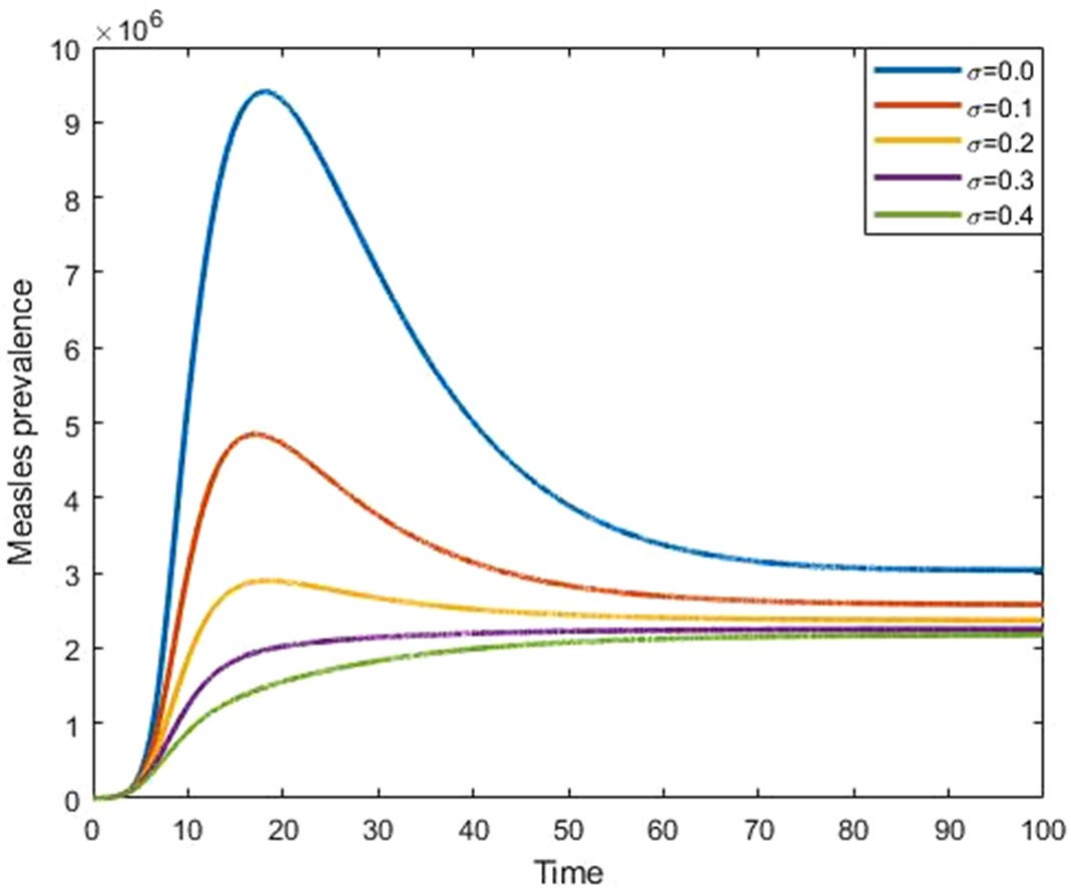
Impact of second dose vaccine $(\upsigma )$ on measles prevalence.

Figures 9 and 10 show that the effect of progression rate and transmission rate on measles prevalence. From these figures, we observed that the burden of the measles prevalence increase if the progression and transmission rates increase, which means that those have a positive correlation with a measles outbreak. Figures 11 and 12 show that the increases in first and second dose vaccination rates reduce the measles prevalence and reduce the risk of an outbreak.

## Summary and conclusion

This paper has developed and analyzed a compartmental transmission dynamics measles model with double dose vaccination in Bangladesh. We have determined an analytic expression for the basic reproduction number using the next-generation matrix and found that the disease-free equilibrium is locally asymptotically stable if the basic reproduction number is less than one. We have also found that measles disease persists in the community if the basic reproduction number is greater than one. Sensitivity analysis has also been performed to explore the impact of model parameters and findings showed that the spread of the measles disease largely depends on the transmission rate. Therefore, effort should be made to minimize unnecessary transmission with measles infected individuals. However, if we treat early measles infected individuals, it will also reduce transmission from infected person to uninfected person. This study has also highlighted the significance of vaccination in controlling and preventing the spread of measles in the community of Bangladesh. Vaccination in a population is the best way to control an outbreak of measles. Numerical analysis has revealed that vaccination has a negative impact on the prevalence of measles. This finding indicates that the improvement in vaccination dose rate decreases the spread of measles. Therefore, to attain a high level of herd immunity for the disease, mass vaccination exercise should be encouraged to cover most of the population to prevent an outbreak of measles in Bangladesh.

## Data Availability

The datasets produced during the study are available from the corresponding author on reasonable request.
